# Optimal Drug Synergy in Antimicrobial Treatments

**DOI:** 10.1371/journal.pcbi.1000796

**Published:** 2010-06-03

**Authors:** Joseph Peter Torella, Remy Chait, Roy Kishony

**Affiliations:** 1 Department of Systems Biology, Harvard Medical School, Boston, Massachusetts, United States of America; 2 Department of Physics, University of Oxford, Oxford, United Kingdom; 3 School of Engineering and Applied Sciences, Harvard University, Cambridge, Massachusetts, United States of America; University of California San Diego, United States of America

## Abstract

The rapid proliferation of antibiotic-resistant pathogens has spurred the use of drug combinations to maintain clinical efficacy and combat the evolution of resistance. Drug pairs can interact synergistically or antagonistically, yielding inhibitory effects larger or smaller than expected from the drugs' individual potencies. Clinical strategies often favor synergistic interactions because they maximize the rate at which the infection is cleared from an individual, but it is unclear how such interactions affect the evolution of multi-drug resistance. We used a mathematical model of *in vivo* infection dynamics to determine the optimal treatment strategy for preventing the evolution of multi-drug resistance. We found that synergy has two conflicting effects: it clears the infection faster and thereby decreases the time during which resistant mutants can arise, but increases the selective advantage of these mutants over wild-type cells. When competition for resources is weak, the former effect is dominant and greater synergy more effectively prevents multi-drug resistance. However, under conditions of strong resource competition, a tradeoff emerges in which greater synergy increases the rate of infection clearance, but also increases the risk of multi-drug resistance. This tradeoff breaks down at a critical level of drug interaction, above which greater synergy has no effect on infection clearance, but still increases the risk of multi-drug resistance. These results suggest that the optimal strategy for suppressing multi-drug resistance is not always to maximize synergy, and that in some cases drug antagonism, despite its weaker efficacy, may better suppress the evolution of multi-drug resistance.

## Author Summary

The use of antibiotics against bacterial infections has led to the emergence of multi-drug resistant pathogens such as tuberculosis and MRSA. In order to control resistance, clinicians have increasingly turned to multi-antibiotic therapies. The common wisdom is to use combinations of drugs that act synergistically to kill the infection, but the impact of drug synergy on the evolution of resistance is unclear. Using mathematical simulations of an *in vivo* infection model, we asked what level of drug synergy would minimize the risk of multi-drug resistance while preserving the efficacy of treatment. We found that synergy may increase or decrease the risk of multi-drug resistance in a given treatment, depending on infection properties such as mutation rate and the availability of resources. Surprisingly, under conditions of strong competition for resources within the host, we found that maximal synergy—currently favored in clinical settings—can actually increase the risk of multi-drug resistance. Our results identify conditions under which drug synergy exacerbates the problem of multi-drug resistance, and offer guidelines for the selection of drug pairs that suppress it.

## Introduction

As antibiotic-resistant pathogens become more common, clinicians increasingly turn to multi-drug treatment to control infection [1]–[5]. The inhibitory effect of two drugs in combination can be larger or smaller than expected from their individual effects, corresponding to synergistic or antagonistic interactions between the drugs respectively [6]–[9]. Synergistic interactions are usually thought of as advantageous since, for a given amount of drug, they more effectively inhibit the growth of drug-sensitive pathogens. However, *in vitro* studies have suggested that, for the same level of inhibition, more synergistic drug pairs may foster antibiotic resistance [10]–[12]. Antagonistic drug combinations, on the other hand, are less effective at inhibiting drug-sensitive pathogens, but can reduce and even invert the selective advantage of single-drug resistant mutants, causing selection against resistance [13].

These recent observations point to a possible tradeoff in the choice of synergistic versus antagonistic drug combinations with respect to their effects on treating infection and suppressing antibiotic resistance. However, while antagonistic drug combinations increase selection against resistance, and should therefore minimize resistance, they also kill the infection more slowly, giving resistance more time to emerge. Antagonism therefore has two contradicting effects on the evolution of resistance: on one hand, it increases the risk of resistance by decreasing antibiotic inhibition and allowing more time for resistance to evolve; on the other hand, it decreases the risk of resistance by decreasing the selective advantage of single drug resistant mutants. We ask which of these opposing effects is stronger, and therefore which type of drug interaction – synergistic or antagonistic – best prevents the overall chance of emergence of multi-drug resistance.

**Figure 1:**
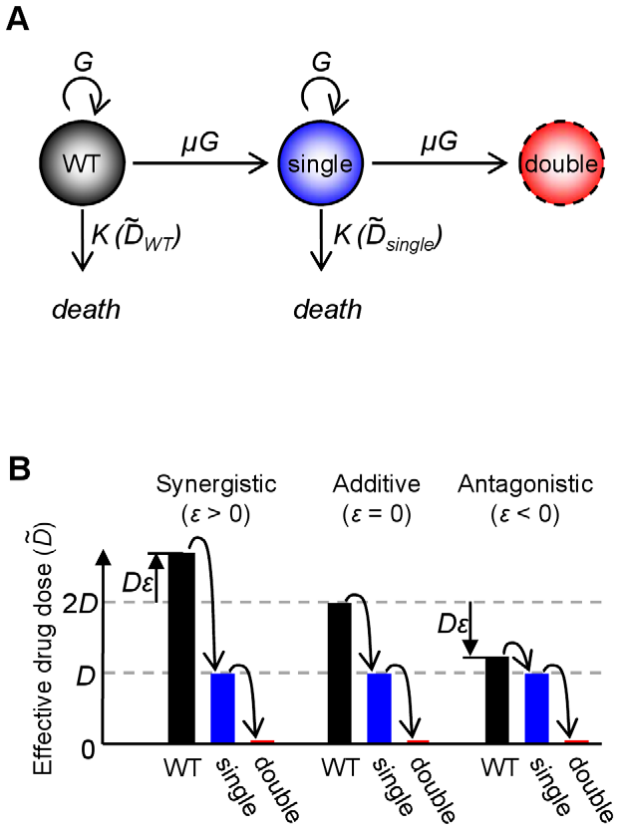
Model of the evolution of resistance in synergistic and antagonistic drug treatments. (A) Graphical representation of model ODEs describing three bacterial subpopulations: the wild-type sensitive to both drugs (black, Eq. 1), single-drug resistant mutants (blue, Eq. 2), and double-drug resistant mutants (red, Eq. 3). Wild-type and single-drug resistant subpopulations grow with rate 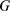 (Eq. 4), mutate with rate 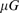 and die with antibiotic killing rates 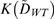 and 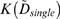, where 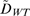 and 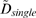 are the effective drug doses they experience, respectively (Eq. 5). We do not model the growth of the double-drug resistant strain, but simply follow the number of such mutants expected to arise via mutation. (B) The wild-type, single-drug resistant and double-drug resistant mutants experience different effective doses, 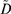, in the multi-drug treatment. The wild-type (black bars) is affected by both the drugs and their interaction, yielding 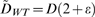, where 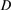 is the dose of each of the drugs A and B (we assume the two drugs are given at the same dose) and 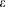 is the level of their interaction (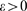, synergistic; 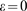, additive; 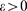, antagonistic). We assume strong resistance, such that resistant mutants are completely unaffected by the drug to which they are resistant; the effective drug dose felt by the single-drug resistant mutant is therefore that of one of the drugs alone, 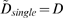, and is independent of 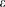 (blue bars have a fixed value). Because double-drug resistant mutants are fully resistant to both antibiotics, they feel an effective dose of 0 (red bars). Increased synergy therefore increases killing of the wild-type, but also increases the selective advantage of the single-drug resistant mutants. Antagonistic drug pairs reduce this selective advantage, and can completely eliminate (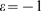) or even invert it (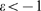).

We frame this problem in the context of a clinical infection, formalizing the two main factors in the success of an antibiotic treatment as “treatment efficacy” and “prevention of multi-drug resistance.” Treatment efficacy is the rate at which the infection is cleared by the treatment, and can be defined as the reciprocal of the time, 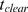, at which the total infection is eliminated, 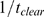. Prevention of multi-drug resistance is defined as the reciprocal of the number of double-drug resistant mutants expected to arise during the course of treatment, 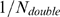. In real infections, multi-drug resistance can arise either through a single mutation conferring cross-resistance to both drugs simultaneously, or through the sequential acquisition of mutations conferring resistance to each drug individually [10], [14]–[16]. Furthermore, resistance to a single drug can develop in several small steps or in one large step [15], [17]–[19]. For simplicity, and to emphasize the role of drug interactions, we concentrate here on an idealized case in which resistance to the two-drug combination evolves through sequential acquisition of two spontaneous mutations, each conferring strong resistance specific to one of the two antibiotics (Fig. 1).

We asked what level of drug interaction (ranging from strong synergy to strong antagonism) maximizes treatment efficacy (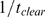) and prevention of multi-drug resistance (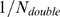). Maximizing 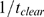 is straightforward: as more synergistic drug pairs have increased killing potency and clear the infection more quickly, maximally synergistic drug pairs should maximize 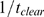 [4], [20]. In attempting to maximize 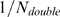, however, the best choice of drug interaction is less clear. Assuming sequential acquisition of resistance to each drug, the rate at which multi-drug resistance arises will depend on the size of the single-drug resistant mutant population. The size of this single-mutant population, in turn, depends on two factors: the rate at which such mutants arise, and their selective advantage over the wild-type. Synergistic drug pairs decrease the first factor because they more quickly kill the source wild-type population from which single mutants arise. However, synergistic drug pairs also increase the second factor: single-drug resistant mutants will have a strong selective advantage in a synergistic treatment because resistance removes both the burden of one drug, and its enhancing effect on the other drug [13] (Fig. 1B). Synergistic drug pairs therefore decrease the rate at which single-drug resistant mutants appear, but increase their selective advantage. Antagonistic drug pairs do the opposite: though they allow a larger number of single-drug resistant mutants to arise, they also diminish the selective advantage of these mutants. The net effect of a given drug interaction on the evolution of multi-drug resistance is therefore not obvious, and requires a quantitative model to determine the overall impact of mutation and selection's countervailing effects.

To better understand how drug interactions affect the risk of multi-drug resistance, we used a population genetic model of microbial infection previously applied to predict single-drug resistance *in vivo* in mice [21], and modified it to account for the sequential acquisition of mutations leading to multi-drug resistance. We used this model to ask what level of drug interaction maximizes treatment efficacy (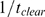) and prevention of multi-drug resistance (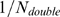).

## Results

### Simple model for the evolution of resistance in multi-drug treatment

We based our model on work by Jumbe *et al.* [21], which investigated a mouse-thigh *P. aeruginosa* infection model [22]–[24] and provided a mathematical model that quantitatively described the relationship between exposure to the fluoroquinolone antibiotic levofloxacin, and changes in drug-susceptible and drug-resistant bacterial subpopulations over time. This mathematical model was successful both in reproducing the observed changes in drug-susceptible and –resistant subpopulations over time, and in predicting the dose of levofloxacin needed to suppress amplification of levofloxacin-resistant (efflux-pump-expressing) mutants. To investigate the effect of antibiotic interactions on treatment efficacy and the prevention of multi-drug resistance in a simple scenario, we modified the Jumbe *et al.* model in four ways: we include a second antibiotic in our model; we assume a constant antibiotic dose; we assume a low hill coefficient, consistent with the mechanisms of a range of antibiotics [25]; and we assume no cost for antibiotic resistance. The consequences of these assumptions are discussed throughout the text.

Our model incorporates treatment with two antibiotics, A and B. It uses a set of ordinary differential equations (ODEs) to follow the population sizes of the drug-sensitive wild-type strain (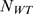), the total single-drug resistant population (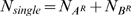; we assume symmetry between drugs A and B such that their respective resistant populations are equal, 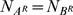), and the expected number of multi-drug resistant mutants (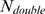) arising over time (Fig. 1A; Methods):
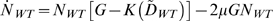
(1)

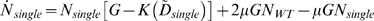
(2)

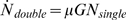
(3)Populations are affected by growth, antibiotic killing and mutation, where 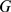, 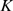 and 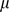 are the growth rate, antibiotic killing rate and frequency of resistance mutations per generation, respectively, and 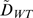 and 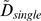 are the effective doses of antibiotic felt by the wild-type and single-drug resistant mutant populations. We assume for simplicity that antibiotic resistance imposes no fitness cost, so that the growth rates of the sensitive and resistant populations are the same. To account for competition for resources, we assume this growth rate is given by the logistic equation,
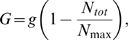
(4)where 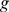 is the maximal growth rate, 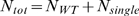 is the total population size, and 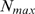 is the maximal carrying capacity (Fig. S1B). This competition for resources was included in the *in vivo* murine infection model [21], and has been observed in or inferred from a range of infections [26], including *S. pneumoniae* [27], and Methicillin-Resistant *S. aureus* (MRSA) [28].

While we assume the growth rates of the wild-type and single-drug resistant mutants are the same, the rates 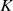 at which they are killed by antibiotic are different and depend on the effective antibiotic dose, 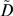, felt by each population:
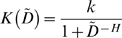
(5)where 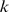 is the maximal killing rate and 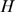 is the Hill coefficient, which determines the steepness of the killing rate as a function of drug dose. In contrast to Jumbe *et al.*, we set 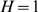, which is representative of many common antibiotics [25], although different values of 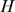 give rise to similar overall model behavior (Fig. S2). The effective drug dose, 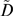, depends on the dosage of the two drugs and on their interaction (Fig. 1B, Text S1, Fig. S1A). For simplicity we assume both drugs are administered at the same dose, 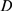, defined in units of their minimum inhibitory concentration (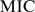), the single-drug dose at which the wild-type death rate equals its growth rate at resource-unlimited conditions: 
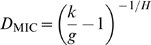
. For the wild-type, the effective dose is the sum of the dosage of the two drugs plus their level of interaction 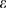: 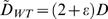. Values of 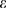 are positive, zero, or negative for synergy, additivity and antagonism, respectively. While in practice the value of 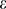 is specific to a given drug pair [29], we treat it as continuous in order to investigate all potential treatment strategies. We assume that single-drug resistant mutants are affected by only one of the drugs, which is reasonable in the case of resistance mechanisms that decrease the intracellular concentration of antibiotic, such as efflux pump expression or enzymatic degradation [11], [13], [30]. The effective dose of single-drug resistant mutants is therefore 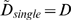, and is independent of 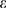 (Fig. 1B). Except where indicated, we set 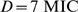 (general model behavior is robust to changes in drug dosage, Fig. S2A), which for an additive drug pair is consistent with the drug dosage used in Jumbe *et al.* [21].

Mutations from wild-type to single-drug resistance, or from single- to double-drug resistance, arise at a rate 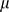 per individual per replication, or 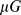 per individual per unit time. Since in any effective treatment the number of double mutants arising is smaller than 1, we do not account for the growth or death of this fractional population, but rather define 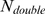 as the integrated number of double mutants generated via mutation during treatment (Eq. 3). Prevention of multi-drug resistance is then defined as 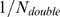.

The model therefore consists of Eqs. 1–5. Parameter values, following Jumbe *et al.* [21], are given in Table S1. Initial conditions for the model are 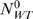, 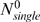, 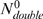 - the population sizes at the onset of treatment. We assume that prior to treatment, the infections have grown from a single cell to the initial population size 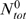 while mutating; while the overall mutation rate is a function of model parameters 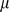, 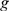 and 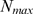 (Eqs. 2, 3), 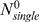 and 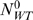 are functions of 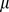 alone: 
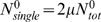
, 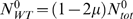. No double-drug resistant mutants are present: 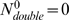. We integrate the ODEs with these initial conditions (Methods) and define 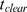 as the time at which the total population size drops below one (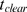 is defined as infinity if the population reaches a non-zero steady state).

### Antibiotic interactions create a saturable tradeoff between treatment efficacy and prevention of multi-drug resistance

**Figure 2:**
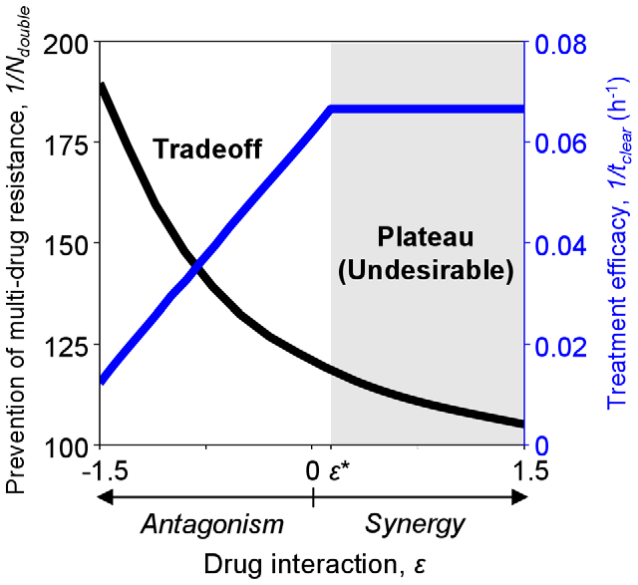
Choice of drug interaction presents a tradeoff between treatment efficacy and prevention of multi-drug resistance. Below a critical level of drug interaction (unshaded region, 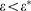), treatment efficacy (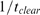, blue) and prevention of multi-drug resistance (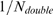, black) exhibit a tradeoff: increased synergy yields higher efficacy, but at the expense of lower resistance prevention. Above 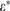, however, efficacy plateaus: increasing synergy beyond this ‘synergy ceiling’ fails to improve treatment efficacy, but continues to diminish resistance prevention (shaded region, 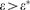).

To determine the impact of drug interaction on treatment outcome, we first looked at the differences in treatment efficacy (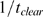) and prevention of multi-drug resistance (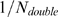) over a range of drug interaction values (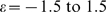) while holding drug dosage fixed (Fig. 2, 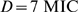). We observed two distinct and robust (Fig. S2) behaviors, depending on whether 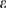 falls above or below a critical value, 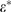 (Fig. 2; for the parameters used, 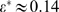).

For 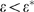, we observed a tradeoff between treatment efficacy and prevention of resistance. In this regime, increasing synergy yields greater 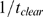 (Fig. 2, unshaded region); this is expected, as increasing the synergistic interaction between the drugs kills the wild-type more quickly. Despite faster infection clearance, however, greater synergy actually decreases 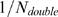; namely, it increases the risk of multi-drug resistance. Conversely, more antagonistic drug pairs increase 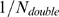, albeit at the expense of reduced efficacy.

This tradeoff between efficacy and prevention of resistance breaks down at a critical threshold, 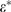, above which increasing synergy no longer increases 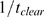, but still decreases 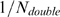 (Fig. 2, shaded region). Above this “synergy ceiling,” further increasing synergy therefore has only undesirable effects, since it increases the risk of multi-drug resistance without increasing efficacy. Optimal drug pairs for treating the given infection must therefore have a level of drug interaction lower than 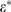 and, due to the tradeoff between 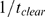 and 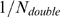, the optimal value of 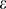 will depend on the relative importance assigned to these two conflicting goals.

### The frequency of resistance mutations determines the “synergy ceiling” 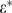


**Figure 3:**
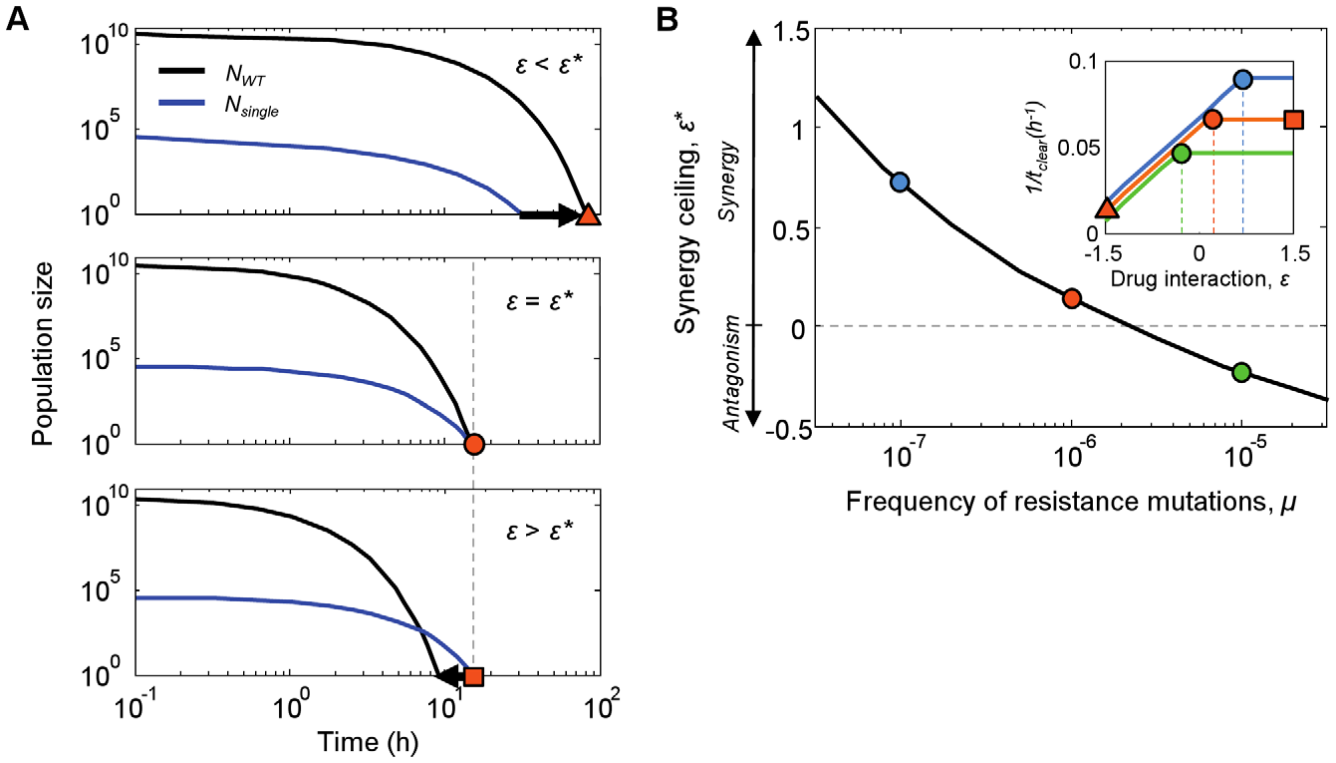
The synergy ceiling 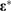 is determined by clearance of the wild-type population before the single-mutant subpopulation. (A) Population sizes of the wild-type (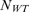, black) and single-drug resistant mutants (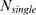, blue) over treatment courses with levels of interaction below, at or above the critical value 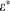 (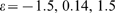). Populations start with sizes 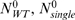 and are killed by antibiotics until they are cleared at times 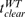 and 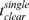 respectively; the overall time of clearance of the infection is simply 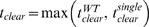 (orange markers). The interaction level 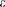 affects the order in which the wild-type and the single-drug resistant subpopulations are eliminated: below the synergy ceiling (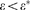, top), the wild-type is eliminated after the single-drug resistant mutant and 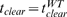; at the synergy ceiling (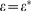, middle), the two populations die simultaneously and 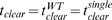; above the synergy ceiling (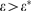, bottom), the single-drug resistant mutant outlives the wild-type, such that 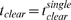. Because increasing 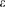 increases the wild-type killing rate but has no effect on the single-mutant killing rate, efficacy increases with 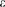 below the synergy ceiling (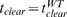), but plateaus at and above it (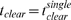; vertical dashed line: notice that 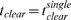 is the same both at and above the synergy ceiling). (B) Increased mutation rates, 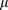, give rise to lower 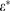. Inset: treatment efficacy, 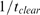, plateaus at lower levels of drug interaction 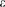 for higher mutation rates (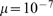, blue; 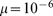, orange; 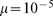, green; blue and green lines are shifted slightly along y-axis for clarity); 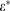 values for each line are indicated by vertical dashed lines, and by circles in the main panel. Orange markers indicate the treatment efficacy achieved for different values of 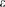 when 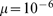, corresponding to the 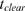 values in panel A.

To understand what determines the level of the synergy ceiling, 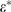, we asked what causes the transition from tradeoff behavior at 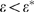, to plateau behavior at 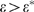 (Fig. 2). Due to the sharp biphasic behavior of efficacy (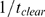) around 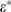, we looked to population time courses to determine how the time of clearance, 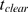, was affected by drug interactions below, at or above 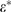 (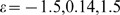, respectively; Fig. 3A). For 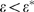 (Fig. 3A, top), the wild-type subpopulation outlives the single-drug resistant mutants and 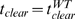. Since wild-type killing is stronger for more synergistic drug pairs, increasing 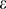 decreases 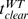, explaining why efficacy increases with 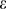 in this region (Fig. 2, unshaded region). For 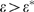 (Fig. 3A; bottom), however, the wild-type is eliminated before the single-mutant population, and 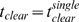. Because the killing rate of the single-drug resistant mutants is independent of 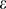, 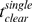 is effectively independent of 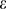, causing 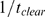 to plateau for 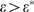 (Fig. 2, shaded region). 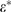 is therefore the level of drug interaction for which wild-type and single-mutant populations are cleared simultaneously (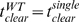; Fig. 3A, middle).

Since 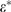 represents the level of drug interaction for which 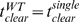, parameters that differentially alter 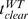 and 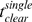 will alter 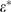. While we found that a number of model parameters had some effect on 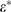 (Fig. S2), the strongest effect was due to changes in the frequency of resistance mutations, 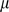. 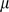 differentially affects 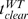 and 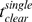 because, although it has virtually no effect on 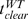, the single-mutant population size at the onset of treatment increases linearly with 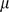, 
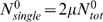
, thereby increasing 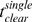. For 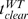 to match this increase in 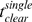, the wild-type killing rate must decrease; namely, 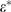 must be reduced. We therefore expected 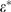 to decrease with increasing 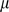 and, indeed, increasing the frequency of resistance mutations gave rise to consistent decreases in 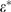 (Fig. 3B). Interestingly, for high frequencies of resistance (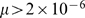) the synergy ceiling 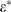 falls below zero (representing an antagonistic drug interaction); in this case mildly synergistic and even additive interactions fall in the undesirable regime where the risk of multi-drug resistance increases without any corresponding gain in treatment efficacy.

### Competition for resources underlies the tradeoff between treatment efficacy and prevention of multi-drug resistance

**Figure 4:**
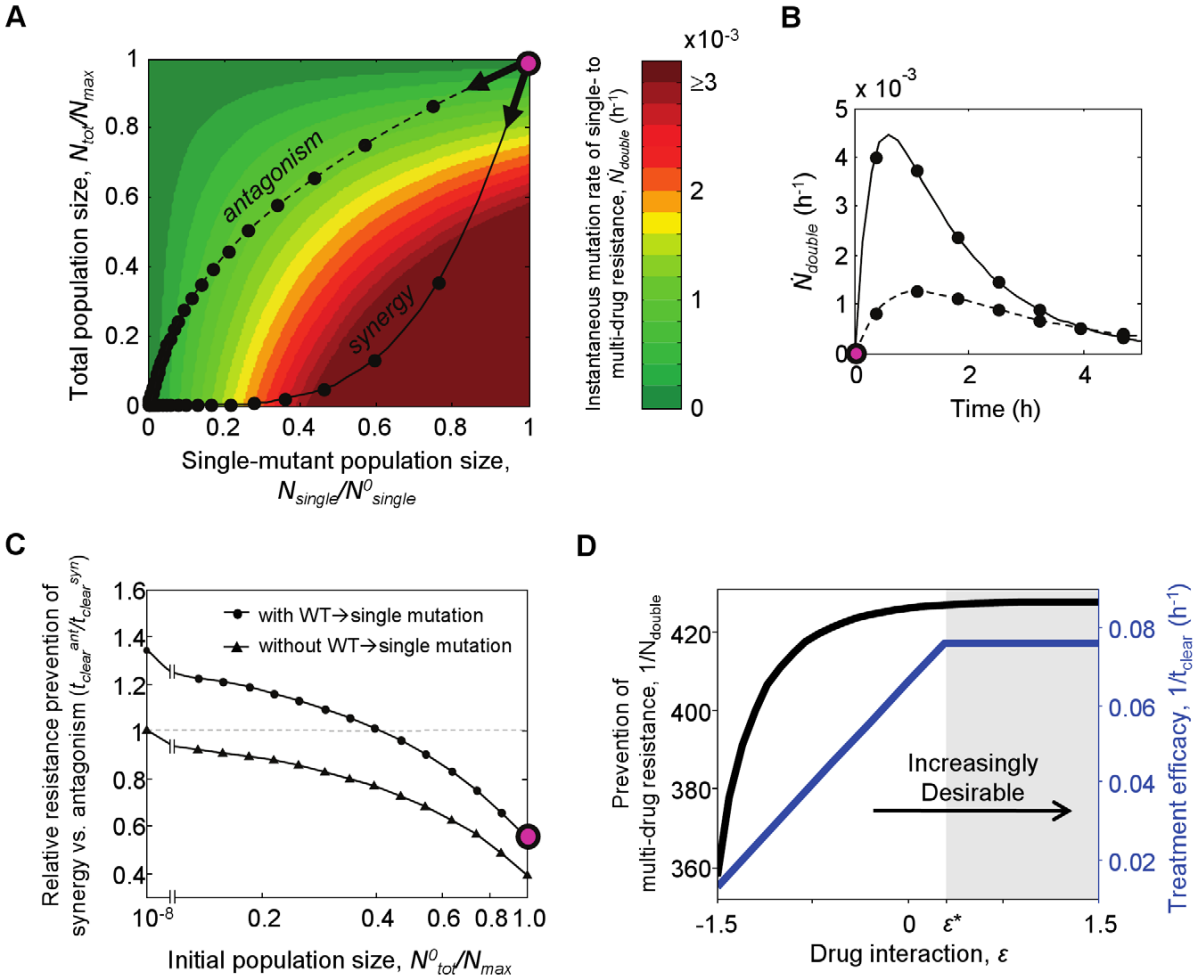
Prevention of multi-drug resistance by drug antagonism depends on resource competition. (A) Heat map of instantaneous rates of double-mutant formation, 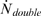, as a function of single-mutant and total population sizes: 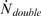 increases with the size of the single-mutant population, and decreases with total population size due to resource competition. Treatment course trajectories for synergistic (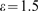, solid line) and antagonistic (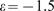, dashed line) drug treatments begin with total initial population size 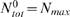 and initial single-mutant population size 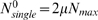 (magenta circle), and move toward the origin as the infection is cleared (black circles indicate 20-minute intervals). The different initial slopes of these trajectories (arrows), determined by the relative fitness of the wild-type and single-drug resistant mutants in synergistic versus antagonistic treatments, lead them to different regions of the heat map: synergistic drug pairs quickly kill the wild-type, relieving resource competition before the single-mutant population is killed and leading to a region with high 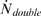 (solid trajectory goes through red region), while antagonistic pairs kill the single mutants before competition is relieved, leading to a region of low 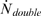 (dashed trajectory goes through green region). (B) The 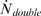 over each treatment plotted as a function of time; black circles indicate 40-minute intervals in this panel. (C) Relative ability of these strongly synergistic and antagonistic drug pairs to prevent multi-drug resistance, 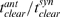, for different initial population sizes (circles). For strong resource competition at the start of treatment (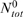 close to 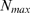), antagonistic drug pairs prevent resistance better than synergistic drug pairs (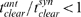). For weak competition, however (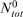 significantly less than 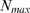), synergistic drug pairs better prevent resistance (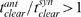). Artificially turning off wild-type to single-drug resistant mutation during treatment (leaving only the single-mutant population that exists at the onset of treatment) eliminates the advantage of synergy over antagonism at low 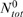 (triangles). (D) When initial population size is low and synergy is advantageous, the tradeoff between treatment efficacy and prevention of multi-drug resistance is eliminated, such that maximally synergistic drug pairs yield both the greatest treatment efficacy and greatest prevention of multi-drug resistance (compare panel C to Fig. 2).

Why does synergy, despite clearing the infection faster, increase the risk of multi-drug resistance (Fig. 2)? Since synergistic drug pairs clear the infection more quickly than antagonistic drug pairs, the rate at which they generate double mutants must also be higher. The overall rate at which double mutants arise, 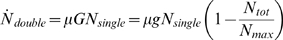 (Eqs. 3, 4), is affected by two variables: it increases with the size of the single-mutant population, 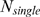, and decreases with total population size, 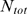, due to the inhibitory effect of resource limitation on growth and mutation (Fig. 4A). The total number of double mutants expected to arise is simply the integral of this instantaneous rate over the treatment course. In order to determine why synergistic treatments increase 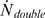, we therefore analyzed the trajectories of synergistic and antagonistic treatments through the space of 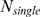 versus 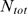 (Fig. 4A; 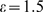, solid line, 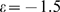, dashed line). The initial slopes of these trajectories (Fig. 4A, arrows) are determined by the relative fitness of the wild-type and single-drug resistant populations under antibiotic treatment. The synergistic treatment selects strongly against the wild-type population, producing a trajectory with a steep slope that drives treatment into a region of high 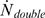 (Fig. 4A, red region); this is because the rapid decrease in wild-type population size relieves competition for resources, creating a window of opportunity in which the still-large single-mutant population can rapidly grow and mutate. Conversely, the antagonistic treatment selects only weakly against the wild-type, producing a trajectory with a shallow slope that skirts the high 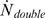 region. Antagonistic drug pairs therefore decrease 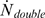 in a competition-dependent fashion: weak killing of the wild-type maintains competition for resources, limiting growth and mutation of the single-drug resistant population until it is eliminated.

It is important to note that resource competition is significant only at the beginning of treatment, when 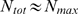. If competition for resources is required for the advantage of antagonism over synergy in preventing resistance, then we should expect a decrease in initial population size to decrease this advantage. To test this prediction, we looked at the relative ability of our representative synergistic (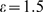) and antagonistic (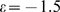) drug pairs to prevent multi-drug resistance, 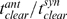, over a range of 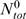 (Fig. 4C, circles; sensitivity to other model parameters is minimal, Fig. S2). Indeed, we found that the advantage of antagonistic drug pairs in preventing resistance (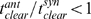, below dashed line) was limited to cases where 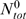 is close to 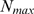. In fact, for 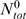 significantly lower than 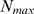, the trend reverses and synergy better prevents resistance (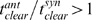, above dashed line). This is because, for low population sizes, resource competition effects are negligible; 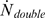 therefore no longer depends on 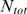, and becomes a function of 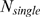 alone. Because synergistic drug pairs better limit 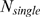 by quickly killing the wild-type population from which single mutants arise, they therefore also better limit multi-drug resistance in cases of weak competition. Indeed, this advantage of synergy disappeared entirely when we artificially turned off wild-type to single-mutant mutation, allowing only those single mutants present at the start of treatment to contribute to 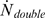 (Fig. 4C, triangles).

Importantly, when competition for resources is weak (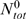 significantly less than 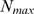, 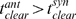), the tradeoff between treatment efficacy and prevention of multi-drug resistance no longer exists (Fig. 4D; compare with Fig. 2). As a result, 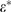 is no longer useful as a “synergy ceiling” because, although drug pairs with 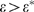 do not further improve 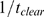, they do improve 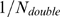. For infections with weak competition, the use of maximally synergistic drug pairs therefore represents the best possible treatment strategy.

## Discussion

We used a population dynamic model of bacterial infection to determine what drug interactions best suppress the emergence of multi-drug resistance. Whereas antagonistic drug pairs kill bacterial populations more slowly, and therefore allow more time for resistance to emerge, they also decrease the selective advantage of resistant mutants. Which of these two opposing effects of antagonism dominates in determining its overall impact on the chance of evolving multi-drug resistance? Framing this problem in the context of a clinical infection, we asked how two measures of treatment outcome, treatment efficacy and prevention of multi-drug resistance, depend on drug interaction.

We found that the optimal drug interaction can be determined primarily as a function of two infection parameters: population size at the outset of treatment, and the frequency of resistance mutations (see summary of our results in Fig. S3). For clinically relevant scenarios where initial population sizes are well below the carrying capacity, competition for resources is weak and synergy, which is typically preferred in clinical settings for its superior treatment efficacy [3], [4], [20], is also expected to best prevent the emergence of multi-drug resistance.

Where resource competition is significant, however, strong synergy may not always be the optimal treatment strategy. Real infections frequently exhibit competition, due either to a scarcity of carbon or iron [26], [31], [32], or saturation of available adhesion sites (e.g. in biofilm formation [33], [34]). In our model, such competition is predicted to give rise to a tradeoff between treatment efficacy and resistance prevention: increased synergy leads to greater efficacy, but at the expense of an increased risk of multi-drug resistance. Importantly, this tradeoff saturates for levels of synergy greater than a critical value 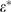, above which greater synergy does not further increase efficacy, but still increases the risk of multi-drug resistance. If the goal is to minimize multi-drug resistance, then choosing drug interactions above this “synergy ceiling” may be counterproductive. This is especially important given the dependence of 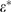 on the frequency of resistance mutations: our model predicts that for infections where resistance rates are high (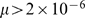) 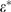 may be negative (antagonistic), favoring the use of antagonistic drug pairs over mildly synergistic or even purely additive antibiotic combinations. Indeed, for the modified Jumbe *et al.* model that we study, 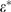 is nearly additive; and while the resistance frequency we use may be an overestimate (Jumbe *et al.* determined this as the rate of all mutations conferring only a 3-fold increase in the MIC), these and higher mutation rates have been identified in human pathogens [35], [36]. Together, the potential for strong competition and high mutation rates in infection suggest that the tradeoff and synergy ceiling behaviors observed in our model – as well as the ability of antagonistic drug pairs to minimize multi-drug resistance – may describe the properties of some clinical infections.

We emphasize that drawing concrete therapeutic conclusions from this study would be beyond its scope. Our model incorporates many simplifying assumptions: we assume 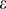 to be a fixed value, although it has been observed to change with both the absolute and relative doses of the antibiotics administered [37], [38]; drug administration and pharmacokinetics are not considered, although they may significantly impact the evolution of resistance [23], [39]–[42]; resistance mutation rates per generation are assumed to be independent of growth and antibiotic-killing rates; and while we consider an idealized case in which multi-drug resistance arises from strong, sequential mutations conferring resistance to each antibiotic, real mutations may confer cross-resistance to both drugs simultaneously, or only partial resistance to a single drug [10], [14], [16]. One consequence of partial resistance is antibiotic killing of drug-resistant mutants for drug interactions above 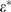; while for strong resistance this killing would be minimal, weak resistance may allow enough killing to undermine synergy ceiling behavior (Fig. S4). Finally, we note that this model does not consider the impact of host immune defenses, which may substantially impact microbial growth and death rates in clinical infections [43], [44]; whether the influence of host defenses favors the use of some drug combinations over others, however, remains to be seen.

While these caveats indicate the limitations of this simple model and suggest important avenues for future study, our results make a number of novel predictions about the relationship between drug interaction and multi-drug resistance: that there exist conditions under which antagonistic drug pairs may better prevent multi-drug resistance despite their weaker efficacy; that there is a synergy ceiling to how much efficacy can be achieved by modulating drug interaction; and that, below this ceiling, changes in drug interaction may produce a tradeoff between inhibition and multi-drug resistance. By basing our model on a previous experimental model of infection [21], we have identified regions of parameter space in which such behaviors may be relevant in a clinical scenario, and which could be tested in future experimental models of infection. Finally, our model highlights the idea that the optimal choice of drug pair in treating an infection may be contextual: while strongly synergistic drug pairs seem the preferred strategy in scenarios where resource limitation and other forms of competition are negligible, antagonistic drug pairs may best prevent resistance in cases of high mutation rates and strong intra-infection competition. While present therapeutic knowledge generally favors synergistic drug pairs, our work motivates further research into the impact and potential utility of antagonistic interactions both in clinical and in ecological settings.

## Methods

### Model details

Our model consists of 3 ODEs (Eq. 1–3) describing the population sizes of the wild-type and single-drug resistant mutants (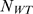, 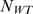), as well as the number of double mutants expected to arise during a treatment course (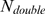). Parameter values for this model include first-order maximal growth (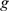) and death rate (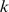) constants, carrying capacity 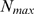 and mutation rate 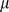 (per individual per generation), which were taken from the *in vivo* murine model investigated in Jumbe *et al.* [21] (Table S1). Initial population sizes (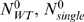) were determined by assuming that, prior to treatment, the infections grew from a single cell to the initial population size 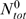 while mutating, such that 
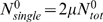
 and 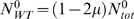; unless otherwise indicated, 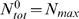. ODEs were solved in MATLAB (Version 7.1, MathWorks, Natick, MA) using a built-in, numerical ODE solver (ODE45). To avoid artifacts associated with using continuous ODEs to describe finite populations, each step was modified with the assumption that the wild-type or single-drug resistant population is eliminated (size decreases to zero) if its size drops below one.

## Supporting Information

Figure S1Models of drug interaction and logistic growth. (A) Model of drug interaction. The effective drug dose for the wild-type strain, 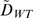, is a function of three variables: the doses of drugs A and B (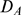, 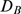) and the interaction parameter 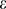 (Text S1). Isoboles of the wild-type effective dose (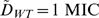), are shown for additive (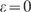, black), synergistic (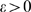, red) and antagonistic (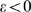, blue) drug pairs. While for additive drug pairs the effective dose is a simple sum of the drugs' individual doses, synergistic or antagonistic drug pairs achieve the same effective dose with smaller or larger drug doses, respectively. All model simulations fall on the dashed line, where drug doses are equal: 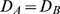. (B) Logistic growth model. As the population size, 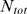, increases, competition causes the growth rate, 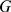, to fall from its maximal value, 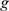, to 0 at the carrying capacity, 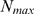 (Eq. 4). Unless otherwise indicated, in model simulations 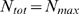 at the outset of treatment (black circle).(0.11 MB TIF)

Figure S2Prevention of resistance, and the synergy ceiling 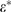, are robust to changes in model parameters. To test the robustness of the model to parameter changes, we varied each parameter independently and measured its effect on both the relative ability of strongly synergistic and antagonistic drug pairs (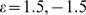) to prevent multi-drug resistance, 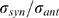 (solid black line), and the level of the synergy ceiling 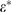 (solid blue line). All lines have undergone 5-point smoothing. Dashed lines indicate the points at which synergistic and antagonistic drug pairs prevent resistance equally well (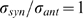, black), or the synergy ceiling is additive (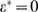, blue). In each panel, those points corresponding to the original set of model parameters are indicated by circles. (A) 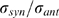 and 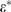 vary little with changes in drug dose *D*, (B) carrying capacity 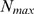, or (C) maximal growth rate 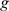. (D) As previously discussed, increases in the frequency of resistance mutations 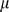 decrease 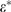 substantially (Fig. 3), while having no significant effect on 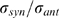. (E) Likewise, increases in the initial population size, 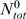, significantly decrease 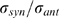 (Fig. 4), but also decrease 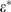. (F) Changes in the Hill coefficient, 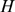, of antibiotic killing had a more complex effect on 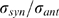 and 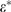: while 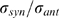 was consistently less than 1 over a wide range of 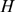 (antagonism better prevents resistance), its magnitude was parabolic with 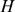, with antagonistic drug pairs having the greatest advantage for 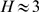. 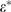 also appeared parabolic with 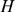 and was lowest (most antagonistic) at 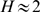. In the limit of large 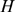, maximal antibiotic killing rates are achieved for both wild-type and single-drug resistant populations, regardless of drug interaction. Synergistic and antagonistic drug pairs therefore fail to differentially impact wild-type killing rates, and 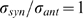 at high 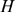 (black line). Furthermore, saturation of killing rates causes 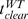 to be greater than 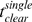 for all values of 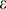; 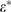 is therefore undefined for 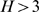 (blue line), though saturation of the wild-type killing rate still causes efficacy to effectively plateau for low values of 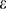.(0.37 MB TIF)

Figure S3Optimal drug interactions as a function of resistance frequency and initial population size. The contour map shows the synergy ceiling, 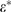, for a given combination of resistance mutation frequency, 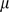, and population size at the start of treatment, 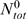. 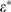 decreases monotonically with increasing 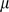 (as in Fig. 3), but is nearly unaffected by 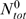. The black line is a single contour above which antagonistic drug pairs prevent multi-drug resistance better than synergistic drug pairs (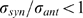). Above this contour, greater synergy increases the chance of multi-drug resistance; the optimal drug interaction must therefore fall below 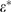, with its specific value depending on the priority assigned to treatment efficacy versus prevention of multi-drug resistance. Below the contour, however (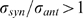), greater synergy decreases the chance of multi-drug resistance; the optimal drug interaction is therefore maximal synergy (region below the black contour is colored dark red), regardless of 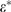. The magenta circle indicates the combination of 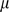 and 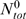 corresponding to the original set of model parameters.(0.10 MB TIF)

Figure S4Partial antibiotic resistance weakens synergy ceiling behavior. In the model we assume strong antibiotic resistance, such that the antibiotic-resistant subpopulation feels the effect of only a single drug; effectively, this makes the MIC of the drug to which it is resistant infinite and produces the familiar synergy ceiling, in which efficacy increases with 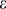 up to a critical level 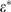 and plateaus above it (blue line, Fig. 2; all lines have been shifted on the vertical axis for clarity). As previously discussed (Fig. 3), this plateau is due to the resistant subpopulation dying after the wild-type when 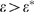. When we weaken the assumption of strong resistance, however (MIC<∞), drug interactions still affect drug-resistant mutants, even when such mutants are killed after the wild-type. This results in efficacy increasing over all 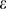, but retaining its characteristic biphasic profile (red, green, orange lines). This biphasic behavior is due to the stronger killing of the wild-type than the resistant mutant, which persists even with only partial resistance. While stronger resistance produces behavior similar to the typical synergy ceiling (MIC increases 100-fold, red line), weaker resistance (MIC increases 4-fold, orange line) yields a still-biphasic curve, but one in which increases in 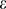 improve efficacy substantially in all cases. The synergy ceiling behavior is therefore most relevant in cases of strong antibiotic resistance.(0.08 MB TIF)

Table S1 Parameters used in this study.
(0.03 MB DOC)

Text S1 (0.02 MB DOC)
