# Correction: Optimal Drug Synergy in Antimicrobial Treatments

**DOI:** 10.1371/annotation/80bc1e50-d623-464f-817f-a5e776b75717

**Published:** 2010-06-11

**Authors:** Joseph Peter Torella, Remy Chait, Roy Kishony

All instances of the symbols "t_ant_clear" and "t_syn_clear" relating to Figures 4 and S2 should instead read "N_ant_double" and "N_syn_double" respectively. Similarly, all instances of "sigma_syn/sigma_ant" relating to Figures S2 and S3 should read "N_ant_double/N_syn_double."

