# Correction: Optimal Drug Synergy in Antimicrobial Treatments

**DOI:** 10.1371/annotation/2505c54a-329b-4553-ad82-cb4b38100ffa

**Published:** 2010-07-09

**Authors:** Joseph Peter Torella, Remy Chait, Roy Kishony

Throughout the captions of Figure S2 and Figure S3 all instances of “sigma_syn/sigma_ant” should read “N_ant_double/N_syn_double.”

