# Correction: Optimal Drug Synergy in Antimicrobial Treatments

**DOI:** 10.1371/annotation/4117feb8-90b6-474f-aba8-0da4aa4b7c21

**Published:** 2010-07-09

**Authors:** Joseph Peter Torella, Remy Chait, Roy Kishony

Throughout the text and figures all instances of the symbols “t_ant_clear” and “t_syn_clear” relating to Figure 4 and Figure S2 should instead read “N_ant_double” and “N_syn_double” respectively.

The correct version of Figure 4 can be found here: 

**Figure:**
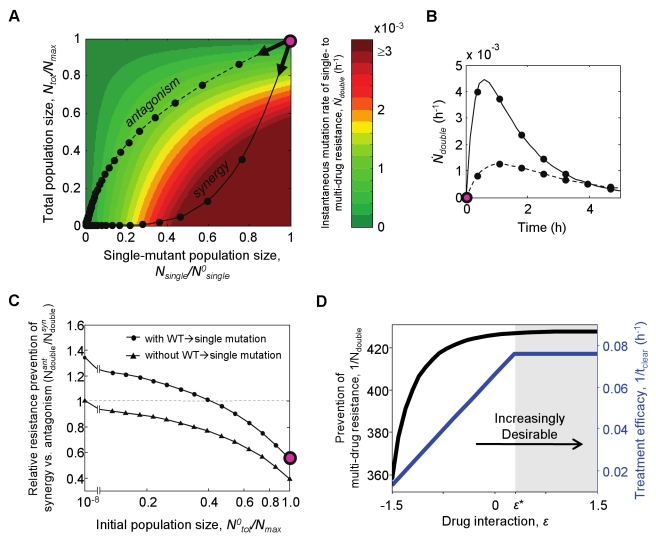


The correct version of Figure S2 can be found here: 
