# Supplementary figures and images for: Optimal Drug Synergy in Antimicrobial Treatments
Source: PLoS Comput Biol. 2010 Jun 3;6(6):e1000796. doi: 10.1371/journal.pcbi.1000796 (PMC2880566; doi:10.1371/journal.pcbi.1000796)

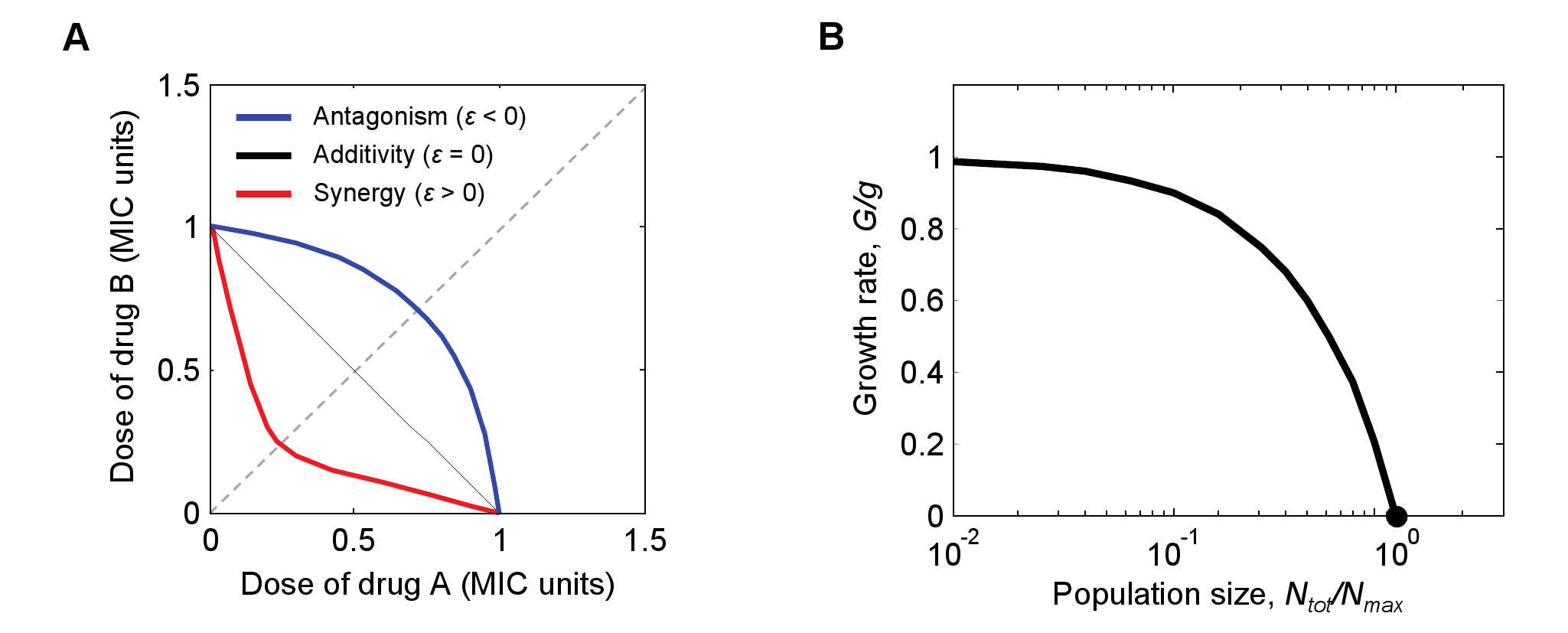

Supplement: Figure S1 — Models of drug interaction and logistic growth. (A) Model of drug interaction. The effective drug dose for the wild-type strain, , is a function of three variables: the doses of drugs A and B (, ) and the interaction parameter (Text S1). Isoboles of the wild-type effective dose (), are shown for additive (, black), synergistic (, red) and antagonistic (, blue) drug pairs. While for additive drug pairs the effective dose is a simple sum of the drugs' individual doses, synergistic or antagonistic drug pairs achieve the same effective dose with smaller or larger drug doses, respectively. All model simulations fall on the dashed line, where drug doses are equal: . (B) Logistic growth model. As the population size, , increases, competition causes the growth rate, , to fall from its maximal value, , to 0 at the carrying capacity, (Eq. 4). Unless otherwise indicated, in model simulations at the outset of treatment (black circle). (0.11 MB TIF) [file pcbi.1000796.s001.tif]

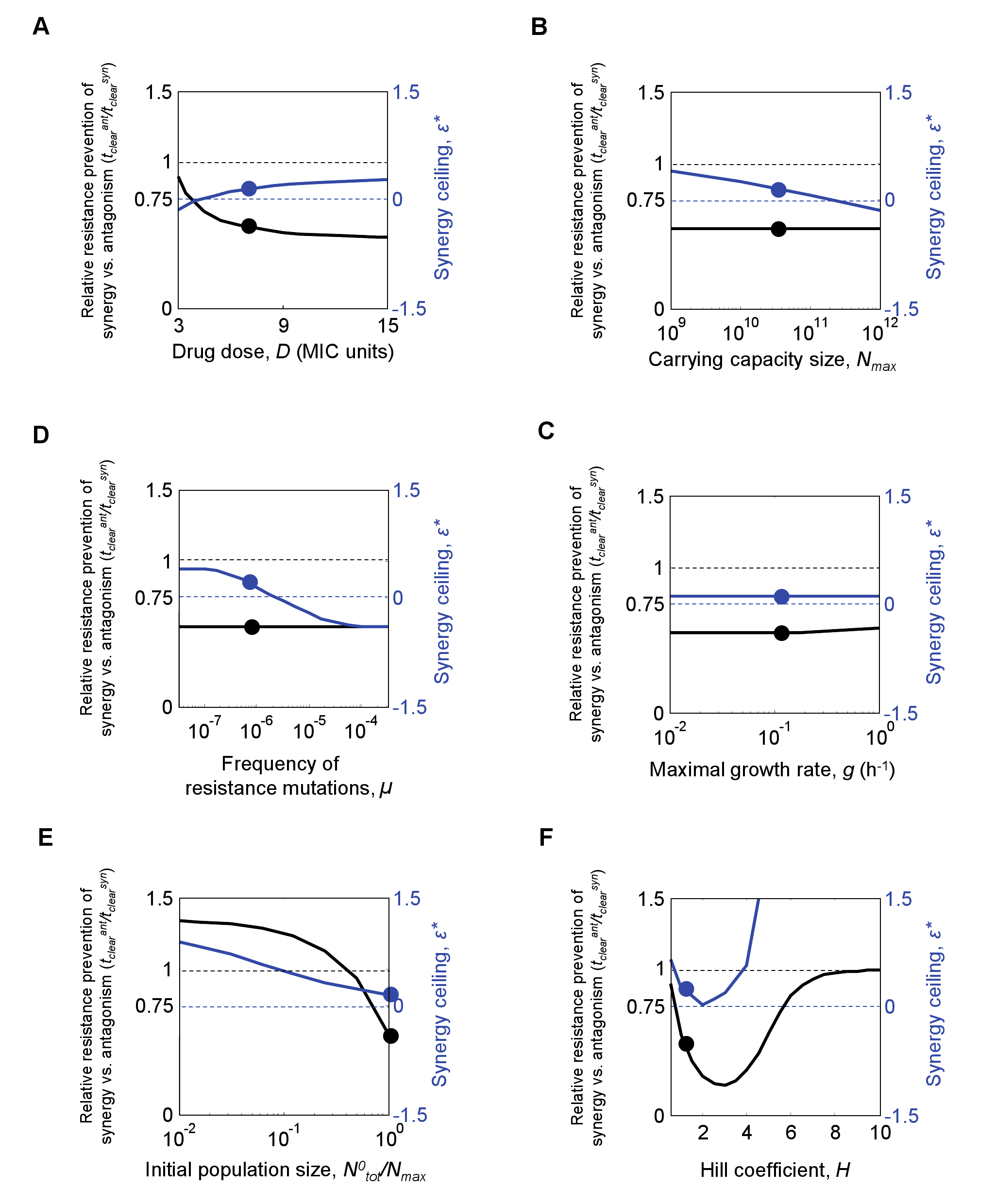

Supplement: Figure S2 — Prevention of resistance, and the synergy ceiling , are robust to changes in model parameters. To test the robustness of the model to parameter changes, we varied each parameter independently and measured its effect on both the relative ability of strongly synergistic and antagonistic drug pairs () to prevent multi-drug resistance, (solid black line), and the level of the synergy ceiling (solid blue line). All lines have undergone 5-point smoothing. Dashed lines indicate the points at which synergistic and antagonistic drug pairs prevent resistance equally well (, black), or the synergy ceiling is additive (, blue). In each panel, those points corresponding to the original set of model parameters are indicated by circles. (A) and vary little with changes in drug dose D, (B) carrying capacity , or (C) maximal growth rate . (D) As previously discussed, increases in the frequency of resistance mutations decrease substantially (Fig. 3), while having no significant effect on . (E) Likewise, increases in the initial population size, , significantly decrease (Fig. 4), but also decrease . (F) Changes in the Hill coefficient, , of antibiotic killing had a more complex effect on and : while was consistently less than 1 over a wide range of (antagonism better prevents resistance), its magnitude was parabolic with , with antagonistic drug pairs having the greatest advantage for . also appeared parabolic with and was lowest (most antagonistic) at . In the limit of large , maximal antibiotic killing rates are achieved for both wild-type and single-drug resistant populations, regardless of drug interaction. Synergistic and antagonistic drug pairs therefore fail to differentially impact wild-type killing rates, and at high (black line). Furthermore, saturation of killing rates causes to be greater than for all values of ; is therefore undefined for (blue line), though saturation of the wild-type killing rate still causes efficacy to effectively plateau for low values of . (0.37 MB [file pcbi.1000796.s002.tif]

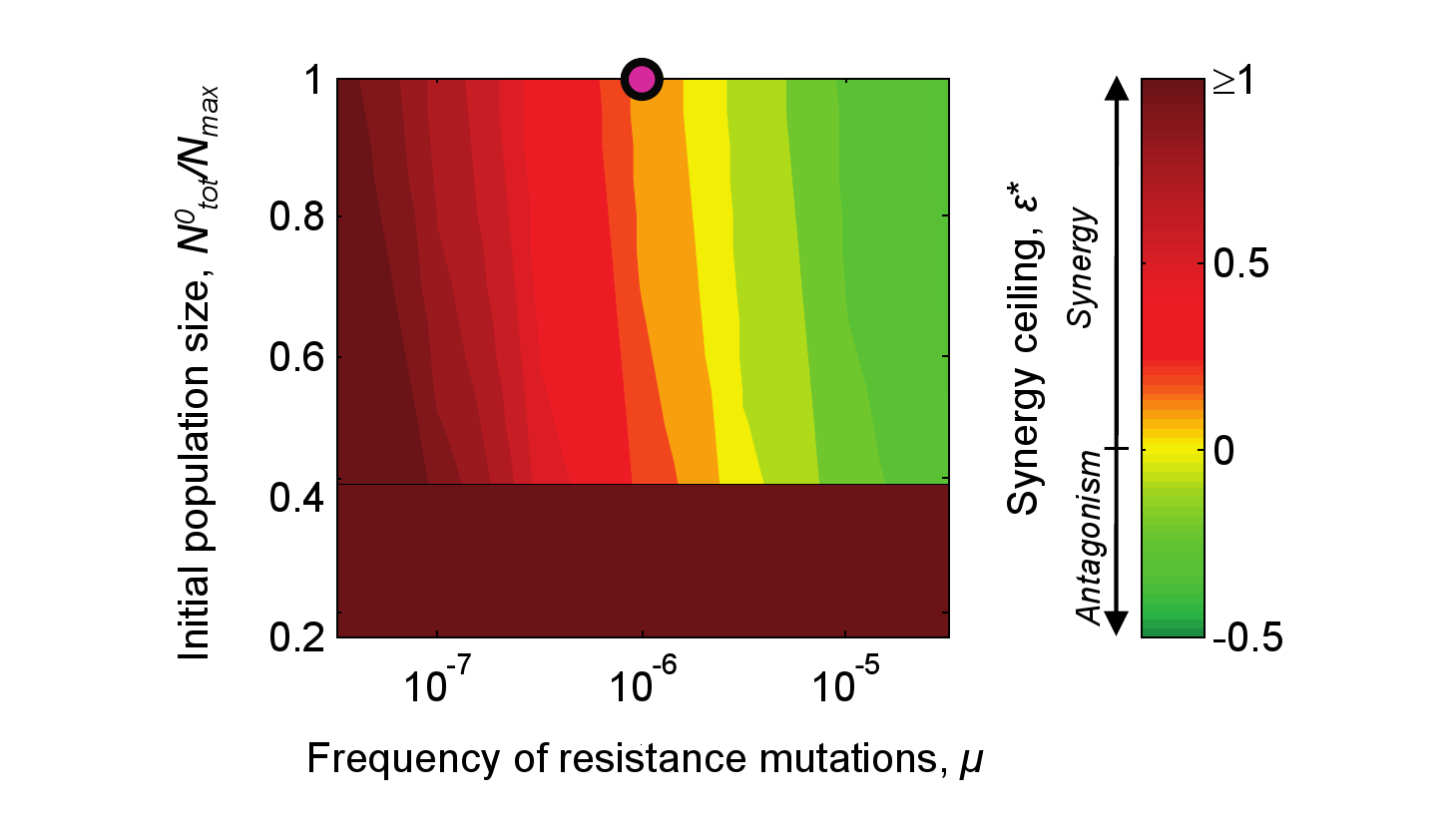

Supplement: Figure S3 — Optimal drug interactions as a function of resistance frequency and initial population size. The contour map shows the synergy ceiling, , for a given combination of resistance mutation frequency, , and population size at the start of treatment, . decreases monotonically with increasing (as in Fig. 3), but is nearly unaffected by . The black line is a single contour above which antagonistic drug pairs prevent multi-drug resistance better than synergistic drug pairs (). Above this contour, greater synergy increases the chance of multi-drug resistance; the optimal drug interaction must therefore fall below , with its specific value depending on the priority assigned to treatment efficacy versus prevention of multi-drug resistance. Below the contour, however (), greater synergy decreases the chance of multi-drug resistance; the optimal drug interaction is therefore maximal synergy (region below the black contour is colored dark red), regardless of . The magenta circle indicates the combination of and corresponding to the original set of model parameters. (0.10 MB TIF) [file pcbi.1000796.s003.tif]

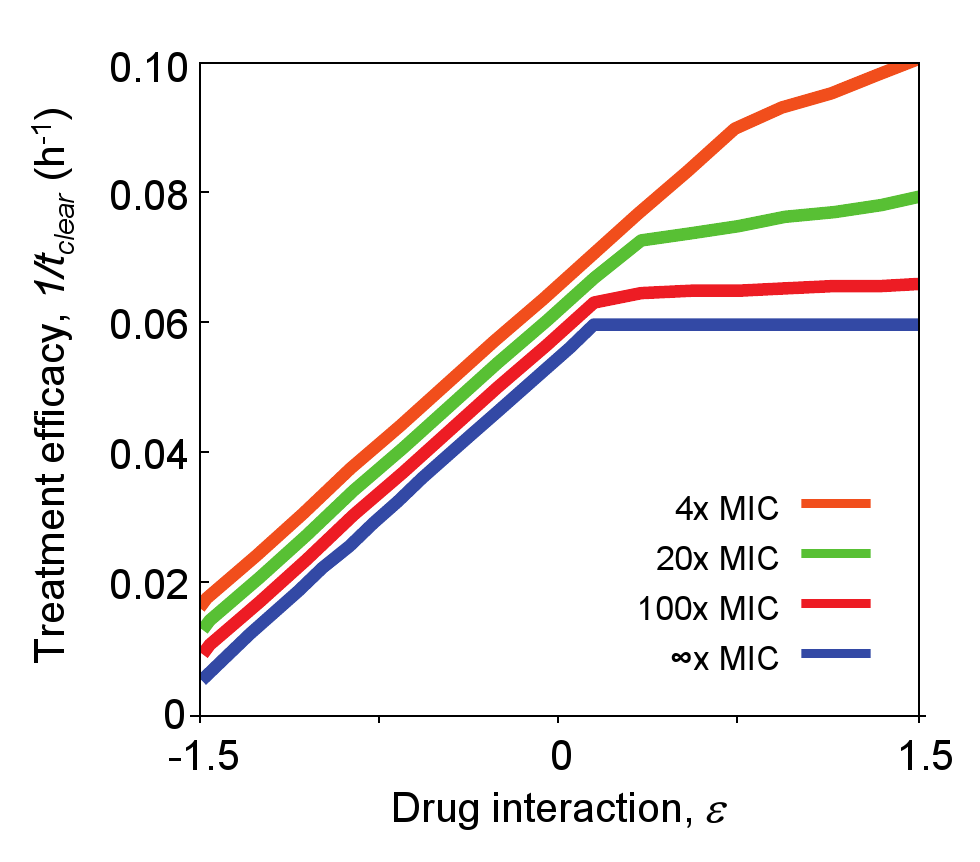

Supplement: Figure S4 — Partial antibiotic resistance weakens synergy ceiling behavior. In the model we assume strong antibiotic resistance, such that the antibiotic-resistant subpopulation feels the effect of only a single drug; effectively, this makes the MIC of the drug to which it is resistant infinite and produces the familiar synergy ceiling, in which efficacy increases with up to a critical level and plateaus above it (blue line, Fig. 2; all lines have been shifted on the vertical axis for clarity). As previously discussed (Fig. 3), this plateau is due to the resistant subpopulation dying after the wild-type when . When we weaken the assumption of strong resistance, however (MIC<∞), drug interactions still affect drug-resistant mutants, even when such mutants are killed after the wild-type. This results in efficacy increasing over all , but retaining its characteristic biphasic profile (red, green, orange lines). This biphasic behavior is due to the stronger killing of the wild-type than the resistant mutant, which persists even with only partial resistance. While stronger resistance produces behavior similar to the typical synergy ceiling (MIC increases 100-fold, red line), weaker resistance (MIC increases 4-fold, orange line) yields a still-biphasic curve, but one in which increases in improve efficacy substantially in all cases. The synergy ceiling behavior is therefore most relevant in cases of strong antibiotic resistance. (0.08 MB TIF) [file pcbi.1000796.s004.tif]
