# Supplementary material for: Optimal Drug Synergy in Antimicrobial Treatments
Source: PLoS Comput Biol. 2010 Jun 3;6(6):e1000796. doi: 10.1371/journal.pcbi.1000796 (PMC2880566; doi:10.1371/journal.pcbi.1000796)
Supplement: Table S1 — Parameters used in this study. (0.03 MB DOC) [file pcbi.1000796.s005.doc]

**Table S1.** Parameters used in this study, from Jumbe *et al.* (21).

| *N_max_* | 3.6 x 10^10^ |
| --- | --- |
| *µ* | 10^-6^ per individual per generation |
| *k* | 94 h^-1^ |
| *g* | 0.117 h^-1^ |
