# Supplementary material for: Optimal Drug Synergy in Antimicrobial Treatments
Source: PLoS Comput Biol. 2010 Jun 3;6(6):e1000796. doi: 10.1371/journal.pcbi.1000796 (PMC2880566; doi:10.1371/journal.pcbi.1000796)
Supplement: Text S1 — (0.02 MB DOC) [file pcbi.1000796.s006.doc]

$\tilde{D}_{WT}=D_{A}+D_{B}+\varepsilon\frac{D_{A}D_{B}\sqrt{2}}{\sqrt{D_{A}^{2}+D_{B}^{2}}}$Text S1
