# Supplementary figures and images for: Correction: Optimal Drug Synergy in Antimicrobial Treatments
Source: PLoS Comput Biol. 2010 Jul 9;6(7):10.1371/annotation/4117feb8-90b6-474f-aba8-0da4aa4b7c21. doi: 10.1371/annotation/4117feb8-90b6-474f-aba8-0da4aa4b7c21 (PMC2909090; doi:10.1371/annotation/4117feb8-90b6-474f-aba8-0da4aa4b7c21)

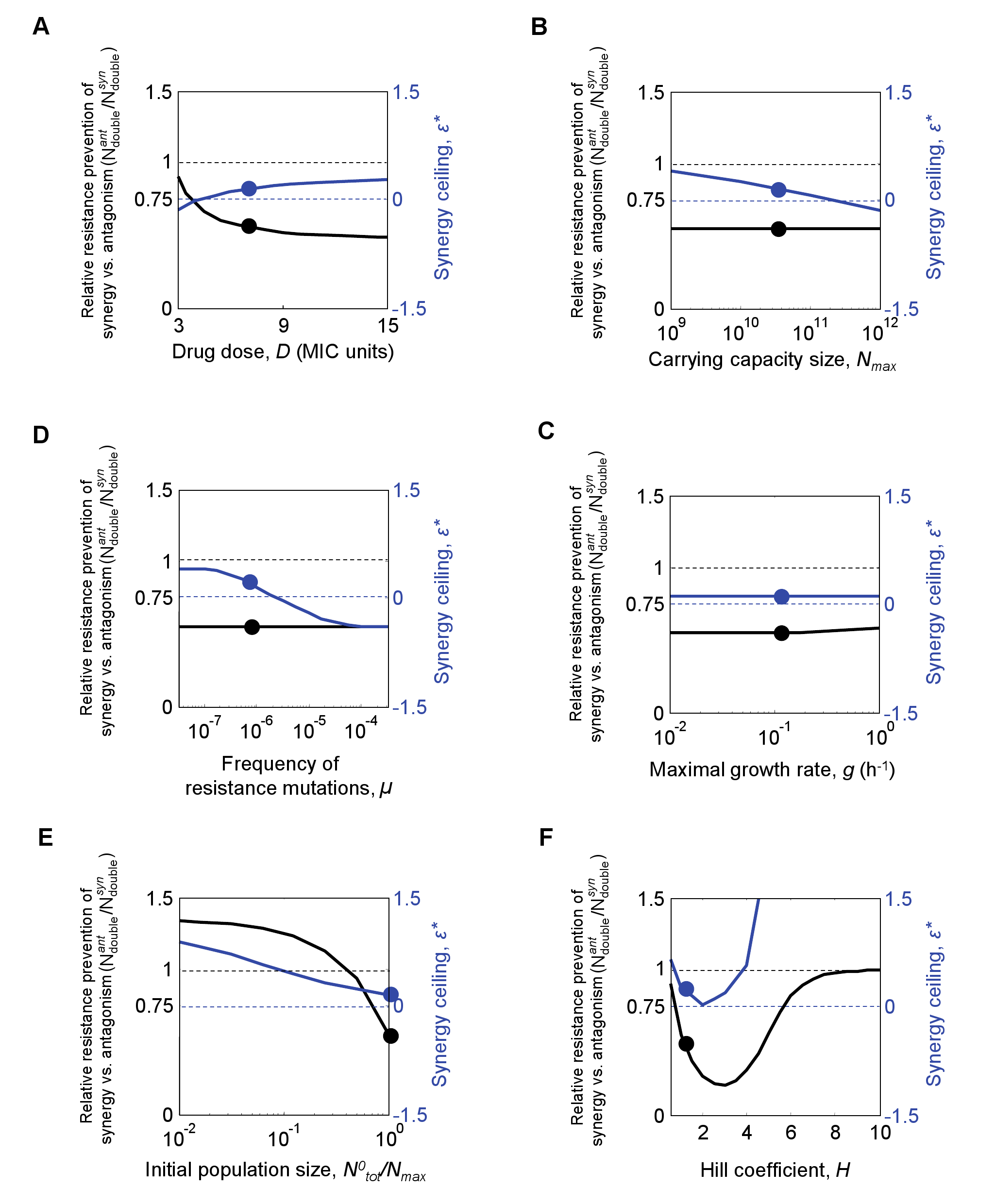

Supplement: Supplementary file 1 [file pcbi.4117feb8-90b6-474f-aba8-0da4aa4b7c21.s001.tif]
